# Thin-slice reverse encoding distortion correction DWI facilitates visualization of non-functioning pituitary neuroendocrine tumor (PitNET)/pituitary adenoma and surrounding normal structures

**DOI:** 10.1186/s41747-024-00430-8

**Published:** 2024-03-07

**Authors:** Shuichi Ito, Sachi Okuchi, Yasutaka Fushimi, Sayo Otani, Krishna Pandu Wicaksono, Akihiko Sakata, Kanae Kawai Miyake, Hitomi Numamoto, Satoshi Nakajima, Hiroshi Tagawa, Masahiro Tanji, Noritaka Sano, Hiroki Kondo, Rimika Imai, Tsuneo Saga, Koji Fujimoto, Yoshiki Arakawa, Yuji Nakamoto

**Affiliations:** 1 Department of Diagnostic Imaging and Nuclear Medicine, Graduate School of Medicine, Kyoto University, 54 Shogoin Kawaharacho, Sakyoku, Kyoto, 606-8507 Japan; 2 Department of Advanced Imaging in Medical Magnetic Resonance, Graduate School of Medicine, Kyoto University, 54 Shogoin Kawaharacho, Sakyoku, Kyoto, 606-8507 Japan; 3 Department of Neurosurgery, Graduate School of Medicine, Kyoto University, 54 Shogoin Kawaharacho, Sakyoku, Kyoto, 606-8507 Japan; 4 MRI Systems Division, Canon Medical Systems Corporation, 1385 Shimoishigami, Otawara, 324-8550 Japan

**Keywords:** Artifacts, Diffusion magnetic resonance imaging, Echo-planar imaging, Neuroendocrine tumors, Pituitary neoplasms

## Abstract

**Background:**

To evaluate the clinical usefulness of thin-slice echo-planar imaging (EPI)-based diffusion-weighted imaging (DWI) with an on-console distortion correction technique, termed *reverse encoding distortion correction* DWI (RDC-DWI), in patients with non-functioning pituitary neuroendocrine tumor (PitNET)/pituitary adenoma.

**Methods:**

Patients with non-functioning PitNET/pituitary adenoma who underwent 3-T RDC-DWI between December 2021 and September 2022 were retrospectively enrolled. Image quality was compared among RDC-DWI, DWI with correction for distortion induced by *B*_0_ inhomogeneity alone (B_0_-corrected-DWI), and original EPI-based DWI with anterior-posterior phase-encoding direction (AP-DWI). Susceptibility artifact, anatomical visualization of cranial nerves, overall tumor visualization, and visualization of cavernous sinus invasion were assessed qualitatively. Quantitative assessment of geometric distortion was performed by evaluation of anterior and posterior displacement between each DWI and the corresponding three-dimensional T2-weighted imaging. Signal-to-noise ratio (SNR), contrast-to-noise ratio (CNR), and apparent diffusion coefficient values were measured.

**Results:**

Sixty-four patients (age 70.8 ± 9.9 years [mean ± standard deviation]; 33 females) with non-functioning PitNET/pituitary adenoma were evaluated. In terms of susceptibility artifacts in the frontal and temporal lobes, visualization of left trigeminal nerve, overall tumor visualization, and anterior displacement, RDC-DWI performed the best and B_0_-corrected-DWI performed better than AP-DWI. The right oculomotor and right trigeminal nerves were better visualized by RDC-DWI than by B_0_-corrected-DWI and AP-DWI. Visualization of cavernous sinus invasion and posterior displacement were better by RDC-DWI and B_0_-corrected-DWI than by AP-DWI. SNR and CNR were the highest for RDC-DWI.

**Conclusions:**

RDC-DWI achieved excellent image quality regarding susceptibility artifact, geometric distortion, and tumor visualization in patients with non-functioning PitNET/pituitary adenoma.

**Relevance statement:**

RDC-DWI facilitates excellent visualization of the pituitary region and surrounding normal structures, and its on-console distortion correction technique is convenient. RDC-DWI can clearly depict cavernous sinus invasion of PitNET/pituitary adenoma even without contrast medium.

**Key points:**

• RDC-DWI is an EPI-based DWI technique with a novel on-console distortion correction technique.

• RDC-DWI corrects distortion due to *B*_0_ field inhomogeneity and eddy current.

• We evaluated the usefulness of thin-slice RDC-DWI in non-functioning PitNET/pituitary adenoma.

• RDC-DWI exhibited excellent visualization in the pituitary region and surrounding structures.

• In addition, the on-console distortion correction of RDC-DWI is clinically convenient.

**Graphical Abstract:**

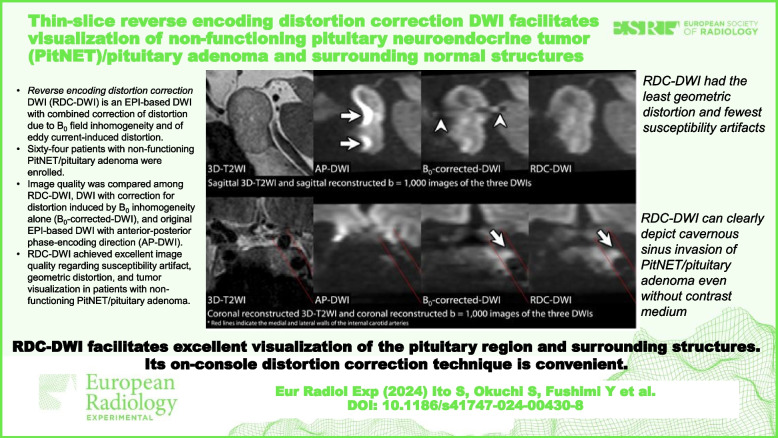

**Supplementary Information:**

The online version contains supplementary material available at 10.1186/s41747-024-00430-8.

## Background

Diffusion-weighted imaging (DWI) is clinically useful for the evaluation of brain tumors [1]. In non-functioning pituitary neuroendocrine tumor (PitNET)/pituitary adenoma, DWI is valuable for assessing tumor consistency [2], prediction of Ki-67 expression [3], prediction of surgical outcome [4], and early detection of recurrence [5]. Echo-planar imaging-based DWI (EPI-DWI) is the most widely used DWI technique but is prone to geometric distortion along the phase-encoding direction due to *B*_0_ field inhomogeneity, especially in the pituitary region [6]. To overcome image degradation due to distortion in the pituitary region, various DWI techniques, including single-shot fast spin-echo DWI [7], line-scan DWI [8], periodically rotated overlapping parallel lines with enhanced reconstruction (PROPELLER)/BLADE DWI [9, 10], reduced-field of view DWI [11], turbo-spin-echo (TSE) DWI [12, 13], and readout-segmented EPI-DWI [14, 15], have been developed. Nevertheless, the long acquisition time and low signal-to-noise ratio (SNR) of these techniques prevent their clinical application [10, 12, 16].

A popular strategy for correction of geometric distortion in EPI-DWI involves acquiring pairs of images with reverse phase-encoding directions, known as “blip-up blip-down” acquisition, to generate an undistorted image. This off-console technique, available as the “*topup*” tool in FMRIB Software Library−FSL (https://fsl.fmrib.ox.ac.uk/fsl/fslwiki/topup), can significantly improve extensive geometric distortion due to *B*_0_ field inhomogeneity using the *b* = 0 s/mm^2^ images with reverse phase-encoding directions [17, 18]. An off-console correction for distortion induced by eddy currents has also been reported [19]. However, off-console techniques such as these require external post-processing and are therefore complicated to use in clinical practice.

Distortion correction techniques for EPI-based DWI have recently been implemented in clinical MRI scanners [20]. A software package termed *reverse encoding distortion correction* DWI (RDC-DWI) has been developed [21, 22], which is a novel on-console correction technique for distortion due to *B*_0_ inhomogeneity and distortion induced by eddy currents. An on-console distortion correction technique of RDC-DWI is more convenient and clinically feasible compared with existing off-console distortion correction tools [17–20].

To the best of our knowledge, no previous study has evaluated the pituitary region on DWI employing an on-console distortion correction technique that uses pairs of images with reverse phase-encoding directions, such as RDC-DWI. This study aims to emphasize the clinical usefulness of thin-slice RDC-DWI by evaluating distortion, susceptibility artifact, and visualization of tumor and surrounding normal structures in patients with non-functioning PitNET/pituitary adenoma, in comparison with DWI with correction for distortion induced by *B*_0_ field inhomogeneity alone (B_0_-corrected-DWI) and original EPI-based DWI with anterior-posterior phase-encoding direction (AP-DWI).

## Methods

### Patient study

**Fig. 1:**
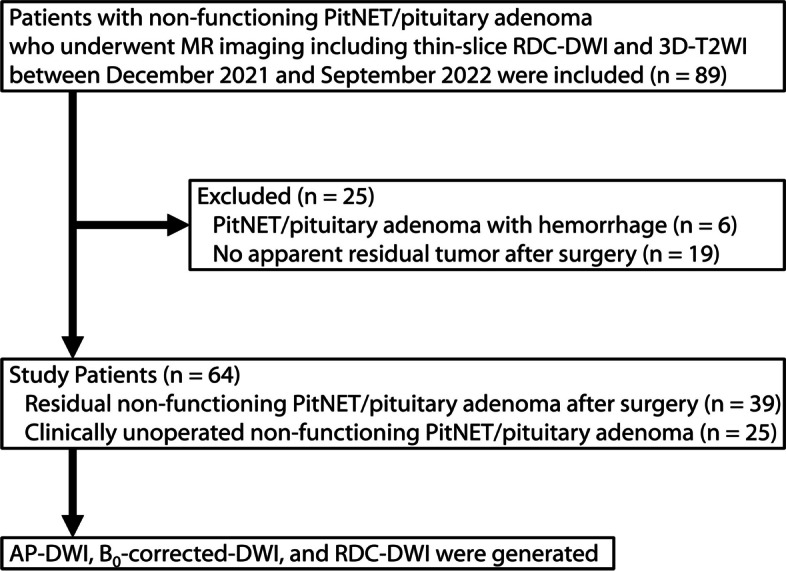
Flow chart of patient enrollment. *3D-T2WI*, Three-dimensional T2-weighted imaging; *DWI*, Diffusion-weighted imaging; *PitNET*, Pituitary neuroendocrine tumor; *RDC-DWI*, Reverse encoding distortion correction DWI

To investigate clinical usefulness of thin-slice RDC-DWI in the pituitary region, patients with non-functioning PitNET/pituitary adenoma were selected because non-functioning PitNET/pituitary adenoma is the most common pituitary tumor. Our institutional review board approved this retrospective study. Informed consent was waived due to its retrospective nature. A flowchart of the study design and patient inclusions is shown in Fig. 1. Eighty-nine consecutive patients (mean age, 68.1 ± 11.4 years [mean ± standard deviation]; 44 females) with non-functioning PitNET/pituitary adenoma who underwent MR imaging, including RDC-DWI with slice thickness of 1.2 mm and three-dimensional T2-weighted imaging (3D-T2WI), between December 2021 and September 2022 were included. Patients were excluded if they met the following exclusion criteria: (a) PitNET/pituitary adenoma with hemorrhage and (b) no apparent residual tumor after surgery.

### Phantom study

To evaluate the reproducibility and accuracy of RDC-DWI, apparent diffusion coefficient (ADC) values were compared among the three DWIs (AP-DWI, B_0_-corrected-DWI, and RDC-DWI) and the theoretical values using a Quantitative Imaging Biomarkers Alliance−QIBA DWI phantom (Model 128, High Precision Devices, Inc., Boulder, USA). The phantom contains 13 vials filled with 30 mL of polymerpolyvinylpyrrolidone (PVP) in an aqueous solution, in six concentrations of PVP (0%, 10%, 20%, 30%, 40%, and 50%) corresponding to the theoretical ADC values specified by the manufacturer. The phantom was used with the temperature maintained between -0.20 and 0.20 °C (ice water bath).

### MRI parameters

All studies were performed using a 3-T MR (Vantage Centurian, Canon Medical Systems Corporation, Otawara, Japan) with a 32-channel head coil. The same technical parameters were used for the phantom study and human study. The imaging parameters for thin-slice RDC-DWI and 3D-T2WI are shown in Table 1. In addition, the *b* = 0 s/mm^2^ images were scanned once for each phase-encoding direction and the *b* = 1,000 s/mm^2^ images were scanned eight times for each phase-encoding direction with simultaneous motion probing gradients (MPGs) in three orthogonal directions.

**Table 1:** The imaging parameters for RDC-DWI and 3D-T2WI

Parameter	RDC-DWI	3D-T2WI
Image acquisition	Axial	Sagittal
*b* value (s/mm^2^)	0, 1,000	−
Repetition time (ms)	3,850	2,800
Echo time (ms)	65	80
Flip angle (degree)	90	90
Field of view (mm^2^)	220 × 220	220 × 220
Voxel size (mm^3^)	0.7 × 0.7 × 1.2	0.34 × 0.34 × 0.8
Number of slices	35	220
Band width (Hz/pixel)	1,563	488
Number of acquisitions	2 (1st, AP direction; 2nd, PA direction)	1
Parallel imaging acceleration factor	3 ×	3 × 2
Acquisition time (min:s)	4:15	4:29

*3D-T2WI* Three-dimensional T2-weighted imaging, *AP* Anterior-posterior, *DWI* Diffusion-weighted imaging, *PA* Posterior-anterior, *RDC-DWI* Reverse encoding distortion correction DWI

### RDC-DWI

RDC-DWI is an on-console distortion correction technique used in EPI-DWI. It corrects distortion induced by eddy currents by using the *b* = 1,000 s/mm^2^ images with reverse phase-encoding direction after application of MPGs in addition to correcting distortion due to *B*_0_ field inhomogeneity by using the *b* = 0 s/mm^2^ images with reverse phase-encoding directions.

### Generation of AP-DWI, B_0_-corrected-DWI, and RDC-DWI

**Fig. 2:**
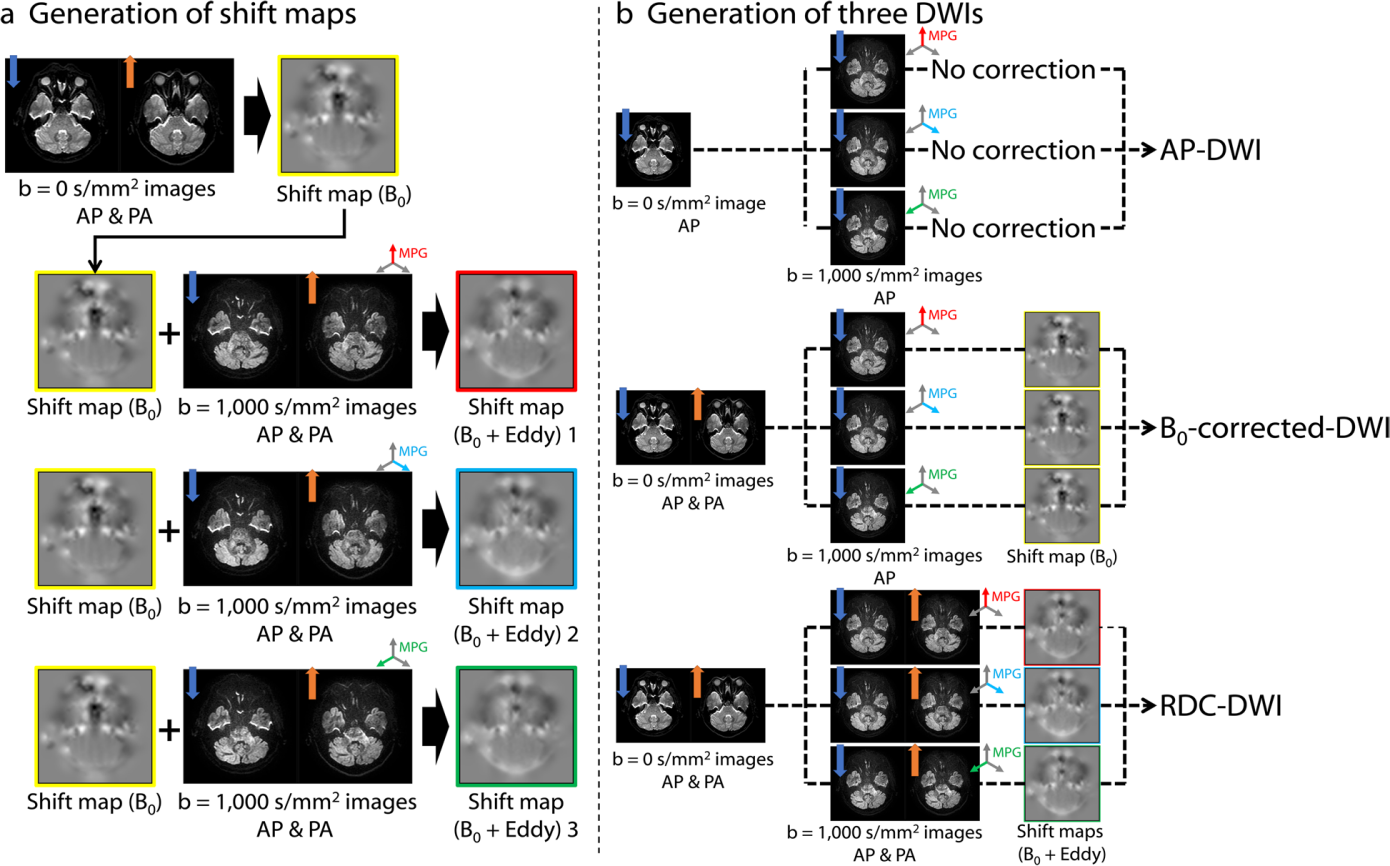
Schematic image of generation of shift maps (**a**) and generation of three DWIs: AP-DWI, B_0_-corrected-DWI, and RDC-DWI (**b**). Shift map (B_0_) is generated using the *b* = 0 s/mm^2^ images with anterior-posterior (AP) and posterior-anterior (PA) phase-encoding directions (**a**, yellow). Shift maps (B_0_ + Eddy) are generated using the shift map (B_0_) and the *b* = 1,000 s/mm^2^ images with AP and PA phase-encoding directions after application of three orthogonal motion probing gradients (MPGs) (**a**, red, blue, and green). AP-DWI is an original EPI-based DWI without distortion correction (**b**, top). B_0_-corrected-DWI is generated using the *b* = 0 s/mm^2^ images with both phase-encoding directions (AP and PA), the *b* = 1,000 s/mm^2^ images with AP phase-encoding direction, and the shift map (B_0_) (**b**, middle). RDC-DWI was generated using the *b* = 0 s/mm^2^ images with both phase-encoding directions (AP and PA), the *b* = 1,000 s/mm^2^ images with both phase-encoding directions (AP and PA), and the shift maps (B_0_ + Eddy) with MPGs in three directions (**b**, bottom). *AP*, Anterior-posterior; *DWI*, Diffusion-weighted imaging; *EPI*, Echo-planar imaging; *MPG*, Motion probing gradient; *PA*, Posterior-anterior; *RDC-DWI*, Reverse encoding distortion correction DWI

AP-DWI, B_0_-corrected-DWI, and RDC-DWI were generated on-console from the same raw data (Fig. 2). A shift map (B_0_) was generated using the *b* = 0 s/mm^2^ images with AP and PA phase-encoding directions. Shift maps (B_0_ + Eddy) were generated using the shift map (B_0_) and *b* = 1,000 s/mm^2^ images with AP and PA phase-encoding directions after application of MPGs. AP-DWI was generated using the *b* = 0 s/mm^2^ images with AP phase-encoding direction and *b* = 1,000 s/mm^2^ images with AP phase-encoding direction without distortion correction. B_0_-corrected-DWI was generated using the *b* = 0 s/mm^2^ images with AP and PA phase-encoding directions, *b* = 1,000 s/mm^2^ images with AP phase-encoding direction, and shift map (B_0_). RDC-DWI was generated using the *b* = 0 s/mm^2^ images with AP and PA phase-encoding directions, *b* = 1,000 s/mm^2^ images with AP and PA phase-encoding directions, and shift maps (B_0_ + Eddy) for three orthogonal MPG directions. In practice, the on-console RDC-DWI generation processes after image acquisition took approximately 2:10 min:s.

### Image analysis: patient study

#### Qualitative evaluation

The image qualities of *b* = 1,000 s/mm^2^ images of the three DWIs (AP-DWI, B_0_-corrected-DWI, and RDC-DWI) were assessed for susceptibility artifact in the frontal and temporal lobes; anatomic visualization of the optic, oculomotor and trigeminal nerves; and overall tumor visualization using a 5-point Likert scale (0, very poor; 1, poor; 2, fair; 3, good; 4, excellent). Visualization of cavernous sinus invasion was assessed qualitatively using a 3-point Likert scale (0, poor; 1, fair; 2, good). Corresponding 3D-T2WI was used as the reference standard for assessing visualization of cavernous sinus invasion. The clinical course and surgical records were referred to when it was difficult to determine the extent of cavernous sinus invasion diagnosis using only 3D-T2WI. The criteria for image assessment are defined in Supplementary Figs. S1–S4. The evaluation was conducted by three board-certified neuroradiologists (S.Ok., S.Ot., and S.I., with 16, 13, and 8 years of experience in neuroradiology, respectively). The three DWIs were provided in random order, and each reader was blinded to the type of distortion correction method. The majority opinion of the raters was designated as the final value. If the three opinions differed, resolution was obtained by consensus.

#### Quantitative evaluation

**Fig. 3:**
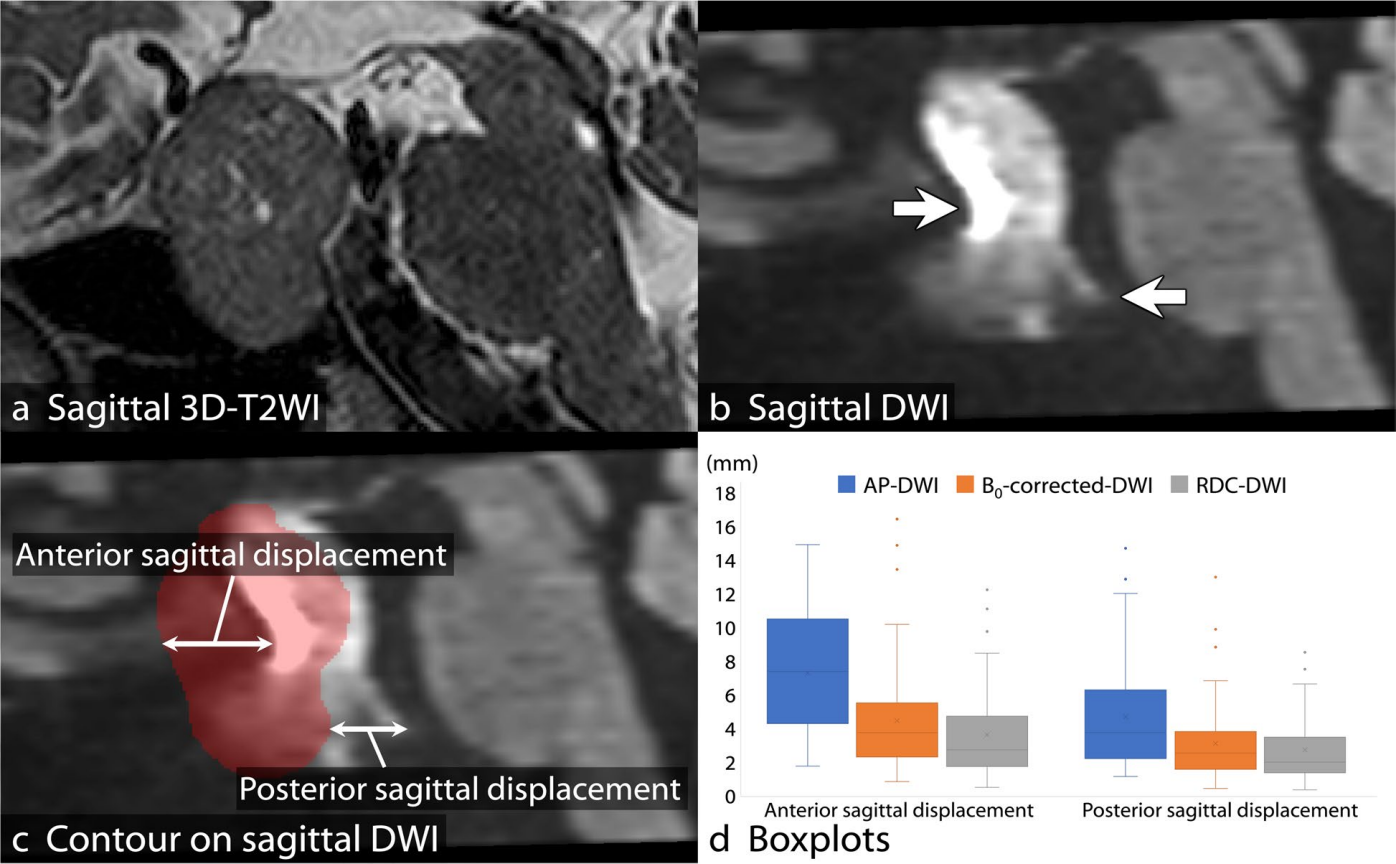
Measurement of anterior and posterior sagittal displacements. The contour of a PitNET/pituitary adenoma on three-dimensional T2-weighted imaging (3D-T2WI) in the sagittal plane (**a**) is superimposed on the corresponding reconstructed sagittal diffusion-weighted imaging (DWI) (**c**). Geometric distortion (**b**, arrows) is determined as anterior and posterior displacements between DWI and the corresponding 3D-T2WI (**c**). The boxplots show results of anterior and posterior sagittal displacements (**d**). Anterior sagittal displacement is the least in RDC-DWI and is significantly lower in B_0_-corrected-DWI than in AP-DWI. Posterior sagittal displacement is significantly lower in RDC-DWI and B_0_-corrected-DWI than in AP-DWI. *3D-T2WI*, Three-dimensional T2-weighted imaging; *AP*, Anterior-posterior; *DWI*, Diffusion-weighted imaging; *EPI*, Echo-planar imaging; *PA*, Posterior-anterior; *PitNET*, Pituitary neuroendocrine tumor; *RDC-DWI*, Reverse encoding distortion correction DWI

Geometric distortion was determined as each of anterior and posterior displacement between each DWI and the corresponding 3D-T2WI in the sagittal plane using ITK-SNAP (http://www.itksnap.org/) (Fig. 3a–c) [23]. To calculate SNR, contrast-to-noise ratio (CNR), and ADC value, regions of interest (ROIs) were placed on the pons and on a solid portion of the PitNET/pituitary adenoma on the *b* = 1,000 s/mm^2^ image and the corresponding ADC map using ImageJ (https://imagej.nih.gov/ij/) (Supplementary Fig. S5). SNR was calculated as SI_pons_/SD_pons_, and CNR was calculated as (SI_lesion_ − SI_pons_)/SD_pons_, where SI_pons_ and SI_lesion_ are mean signal intensity of the pons and the solid portion of the PitNET/pituitary adenoma, respectively, and SD_pons_ is standard deviation of the pons. ROIs that including signal pileup artifacts were excluded for evaluating CNR and ADC value for PitNET/pituitary adenomas.

Evaluation of distortion and ROI measurements were performed by a board-certified radiologist (S.I.) and approved by another board-certified radiologist (S.Ok.).

### Image analysis: phantom study

**Fig. 4:**
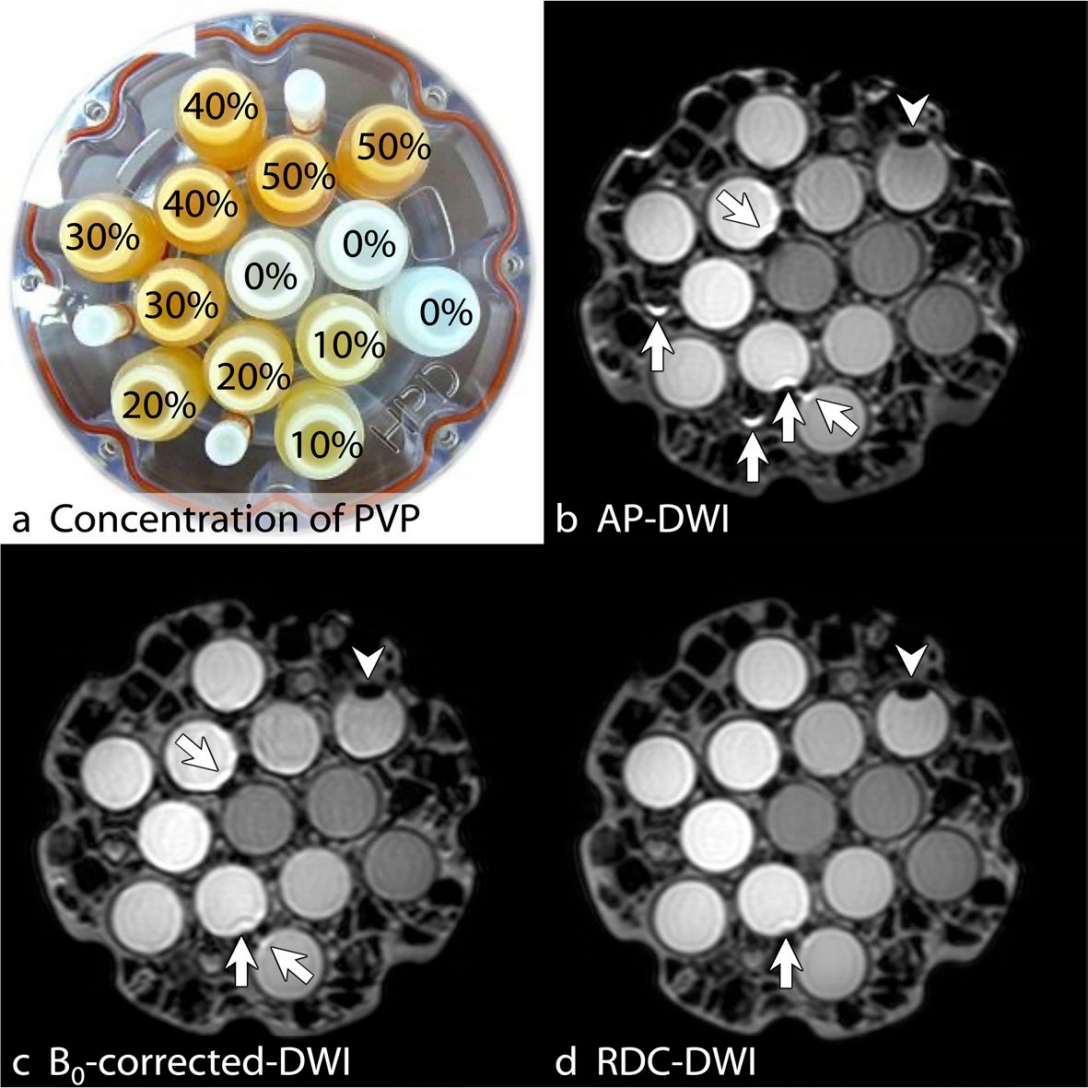
QIBA DWI phantom. Photograph of shows 13 vials containing PVP within the phantom, with PVP concentrations of 0%, 10%, 20%, 30%, 40%, and 50% (**a**). The *b* = 1,000 s/mm^2^ images of the phantom acquired with AP-DWI (**b**), B_0_-corrected-DWI (**c**), and RDC-DWI (**d**) are shown. Compared with AP-DWI and B_0_-corrected-DWI, RDC-DWI shows the least distortion and fewest susceptibility artifacts (**b**, **c**, and **d**, arrows). Air contamination is observed in one of the 50% PVP vials (**b**, **c**, and **d**, arrowheads). *AP*, Anterior-posterior; *DWI*, Diffusion-weighted imaging; *PVP*, Polymerpolyvinylpyrrolidone; *QIBA*, Quantitative Imaging Biomarkers Alliance; *RDC-DWI*, Reverse encoding distortion correction DWI

ROIs of area 212 mm^2^ were drawn on six different slices of the 13 vials with PVP concentrations of 0%, 10%, 20%, 30%, 40%, and 50% (Fig. 4a). The mean ADC values were measured on ADC maps for the three DWIs (AP-DWI, B_0_-corrected-DWI, and RDC-DWI) by a board-certified radiologist (S.I.) using ImageJ. Data of vials with 50% PVP were discarded because of air contamination (Fig. 4b–d).

### Statistical analysis

Interrater reliability for image quality scores measured independently by the three radiologists was evaluated using Fleiss’ *κ* statistics [24]. The calculated *κ* statistic was interpreted as follows: ≤ 0.20, slight agreement; 0.21–0.40, fair agreement; 0.41–0.60, moderate agreement; 0.61–0.80, substantial agreement; and 0.81–1.00, almost perfect agreement. Image quality scores and measured displacements were compared among the three DWIs using the Friedman test followed by pairwise comparisons with Bonferroni correction because the results did not follow a normal distribution. SNR, CNR, and ADC values were also compared among the three DWIs using one-way repeated measures analysis of variance−ANOVA followed by pairwise comparisons with Bonferroni correction because these data followed a normal distribution. A *p*-value less than 0.050 was considered statistically significant.

Fleiss’ *κ* statistics and Friedman test were performed using RStudio Software (version 2022.12.0, RStudio, PBC, Boston, USA), and analysis of variance was performed using MedCalc version 20 (MedCalc Software, Ostend, Belgium).

## Results

### Patient study

#### Participants

Six patients with PitNET/pituitary adenoma with hemorrhage and 19 patients with no apparent residual tumor after surgery were excluded (Fig. 1). The remaining 64 patients (mean age, 70.8 ± 9.9 years [mean ± standard deviation]; 33 females) were evaluated and comprised 25 patients with unoperated clinically non-functioning PitNET/pituitary adenoma and 39 patients with residual non-functioning PitNET/pituitary adenoma after surgery. The demographics of all 64 participants are shown in Table 2.

**Table 2:** Patient demographics

	Number
Total patients	64
Mean age ± standard deviation (years)	70.8 ± 9.9
Male *versus* female	31 *versus* 33
Unoperated clinically non-functioning PitNET/pituitary adenoma	25
Residual non-functioning PitNET/pituitary adenoma after surgery	39
Open surgery	2
Endoscopic surgery	32
Combined surgery	5
Major medical history	
72-year-old man with superficial hemosiderosis	1
71-year-old woman with cerebral aneurysm clip	1
65-year-old man with jugular foramen schwannoma	1
Chiasma/optic nerve compression	37
Chiasma compression	27
Right optic nerve compression	5
Left optic nerve compression	5

*PitNET* Pituitary neuroendocrine tumor

#### Qualitative evaluation

Interrater reliability for qualitative evaluations was moderate to substantial agreement (*κ* = 0.59–0.78) (Supplementary Table S1). In 11 cases of cavernous sinus invasion, the diagnosis was determined with agreement of the three evaluators by also referring to the clinical course and surgical records because of difficulty using only 3D-T2WI.

**Fig. 5:**
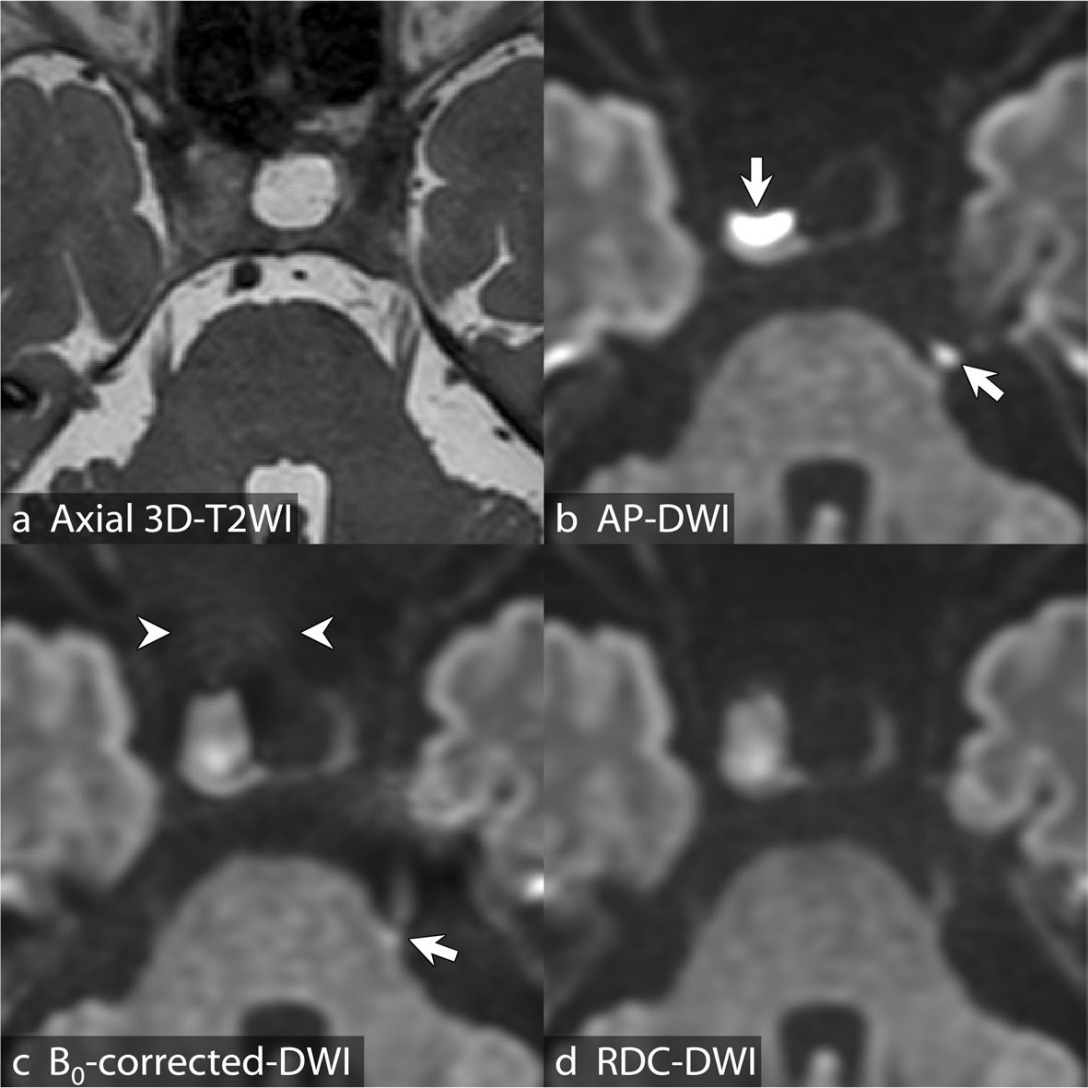
A 68-year-old woman with residual non-functioning PitNET/pituitary adenoma after surgery. Axial 3D-T2WI (**a**), axial *b* = 1,000 s/mm^2^ images of AP-DWI (**b**), B_0_-corrected-DWI (**c**), and RDC-DWI (**d**) are shown. The residual PitNET/pituitary adenoma cannot be clearly distinguished from the right cavernous sinus on axial 3D-T2WI (**a**). There is severe distortion, susceptibility artifact, and signal pileup around the residual PitNET/pituitary adenoma and the left trigeminal nerve on AP-DWI (**b**, arrows). B_0_-corrected-DWI and RDC-DWI depict undistorted residual PitNET/pituitary adenoma without apparent susceptibility artifact (**c** and **d**). Compared with B_0_-corrected DWI, there is less blurring on RDC-DWI, particularly in the sphenoid sinus (**c**, arrowheads), and there are fewer susceptibility artifacts in the left trigeminal nerve (**c**, arrow). *3D-T2WI*, Three-dimensional T2-weighted imaging; *AP*, Anterior-posterior; *DWI*, Diffusion-weighted imaging; *PitNET*, Pituitary neuroendocrine tumor; *RDC-DWI*, Reverse encoding distortion correction DWI

Image quality scores and *p*-values are summarized in Table 3 and Supplementary Fig. S6. Scores for susceptibility artifacts in the frontal and temporal lobes were the best in RDC-DWI (median, 2.0; interquartile range, 2.0–2.0 and 2.0; 2.0–2.0, respectively) and significantly better in B_0_-corrected-DWI (2.0; 1.5–2.0 and 2.0; 2.0–2.0, respectively) than in AP-DWI (1.0; 1.0–1.0 and 1.0; 1.0–2.0, respectively). Scores for overall tumor visualization and anatomical visualization of the left trigeminal nerve were also the best in RDC-DWI (3.0; 3.0–4.0 and 4.0; 3.0–4.0, respectively) and were significantly better in B_0_-corrected-DWI (3.0; 3.0–3.0 and 3.0; 3.0–4.0, respectively) than in AP-DWI (2.0; 2.0–3.0 and 3.0; 3.0–4.0, respectively). Scores for anatomical visualization of the right oculomotor and right trigeminal nerves were significantly better in RDC-DWI (3.0; 2.0–3.0 and 4.0; 3.0–4.0, respectively) than in B_0_-corrected-DWI (3.0; 2.0–3.0 and 3.0; 3.0–3.0, respectively) and AP-DWI (3.0; 2.0–3.0 and 3.0; 3.0–3.0, respectively), but there was no significant difference between B_0_-corrected-DWI and AP-DWI. Scores for visualization of cavernous sinus invasion were significantly better in RDC-DWI (2.0; 2.0–2.0) and B_0_-corrected-DWI (2.0; 2.0–2.0) than in AP-DWI (1.0; 0.0–2.0), but there was no significant difference between RDC-DWI and B_0_-corrected-DWI. Score for anatomical visualization of the left optic nerve was better in B_0_-corrected-DWI (3.0; 3.0–3.0) than in AP-DWI (3.0; 3.0–3.0), but there was no significant difference between RDC-DWI (3.0; 3.0–3.0) and AP-DWI or between RDC-DWI and B_0_-corrected-DWI. Scores for anatomical visualization of the right optic and left oculomotor nerves showed no significant difference among the three DWIs. Representative images of the three DWIs are shown in Fig. 5 and Supplementary Fig. S7.

**Table 3:** Results of qualitative evaluations for AP-DWI, B_0_-corrected-DWI, and RDC-DWI

	AP-DWI	B_0_-corrected-DWI	RDC-DWI	*p*-value
Frontal artifact	1.0 (1.0–1.0)	2.0 (1.5–2.0)	2.0 (2.0–2.0)	< 0.001^a, b, c^
Temporal artifact	1.0 (1.0–2.0)	2.0 (2.0–2.0)	2.0 (2.0–2.0)	< 0.001^a, b^, 0.001^c^
Right optic nerve	3.0 (3.0–3.0)	3.0 (3.0–3.0)	3.0 (3.0–3.0)	0.220^a^, 0.290^b^, 1.000^c^
Left optic nerve	3.0 (3.0–3.0)	3.0 (3.0–3.0)	3.0 (3.0–3.0)	0.003^a^, 0.093^b^, 1.000^c^
Right oculomotor nerve	3.0 (2.0–3.0)	3.0 (2.0–3.0)	3.0 (2.0–3.0)	1.000^a^, 0.037^b^, 0.002^c^
Left oculomotor nerve	3.0 (2.0–3.0)	3.0 (2.0–3.0)	3.0 (3.0–3.0)	1.000^a^, 0.146^b^, 0.071^c^
Right trigeminal nerve	3.0 (3.0–3.0)	3.0 (3.0–3.0)	4.0 (3.0–4.0)	0.085^a^, < 0.001^b, c^
Left trigeminal nerve	3.0 (3.0–4.0)	3.0 (3.0–4.0)	4.0 (3.0–4.0)	0.013^a^, < 0.001^b, c^
Cavernous sinus invasion	1.0 (0.0–2.0)	2.0 (2.0–2.0)	2.0 (2.0–2.0)	< 0.001^a, b^, 0.700^c^
Tumor visualization	2.0 (2.0–3.0)	3.0 (3.0–3.0)	3.0 (3.0–4.0)	< 0.001^a, b, c^

Data are presented as the median (interquartile range) score. Note that susceptibility artifact in the frontal and temporal lobes; anatomic visualization of the optic, oculomotor, and trigeminal nerves; and overall tumor visualization are assessed using a 5-point Likert scale (0, very poor; 1, poor; 2, fair; 3, good; 4, excellent), and visualization of cavernous sinus invasion is assessed using a 3-point Likert scale (0, poor; 1, fair; 2, good)

^a^AP-DWI *versus* B_0_-corrected-DWI

^b^AP-DWI *versus* RDC-DWI

^c^B_0_-corrected-DWI *versus* RDC-DWI

*AP* Anterior-posterior, *DWI* Diffusion-weighted imaging, *RDC-DWI* Reverse encoding distortion correction DWI

#### Quantitative evaluation

Anterior sagittal displacement due to geometric distortion was the least in RDC-DWI (median, 2.8 mm; interquartile range, 1.8–4.7 mm) (*p* < 0.001) and significantly lower in B_0_-corrected-DWI (3.8 mm; 2.4–5.5 mm) than in AP-DWI (7.4 mm; 4.3–10.5 mm) (*p* < 0.001). Posterior sagittal displacement was significantly lower in RDC-DWI (2.0 mm; 1.4–3.5 mm) and B_0_-corrected-DWI (2.6 mm; 1.6–3.9 mm) than in AP-DWI (3.8 mm; 2.3–6.2 mm) (*p* < 0.001). The measured value of posterior sagittal displacement was the least in RDC-DWI. There was no significant difference between RDC-DWI and B_0_-corrected-DWI (*p* = 0.065) (Fig. 3d).

Table 4 lists the SNR, CNR, and ADC values. SNR was the highest in RDC-DWI and was significantly higher in AP-DWI (*p* < 0.001) than in B_0_-corrected-DWI (*p* = 0.001). After excluding 26 patients in whom ROIs contained signal pileup artifacts, CNR and ADC values of the solid portion of PitNET/pituitary adenomas were calculated in 38 patients. Among the three DWIs, CNR was the highest in RDC-DWI, and CNR was significantly higher in RDC-DWI than in B_0_-corrected-DWI (*p* < 0.001). There was no statistically significant difference in CNR between B_0_-corrected-DWI and AP-DWI (*p* = 1.000), or between RDC-DWI and AP-DWI (*p* = 0.094). There was no significant difference in ADC values of the pons or the solid portion of PitNET/pituitary adenomas among the three DWIs.

**Table 4:** SNR, CNR, and ADC values according to DWI type

	AP-DWI	B_0_-corrected-DWI	RDC-DWI	*p*-value
SNR	11.7 ± 2.4	11.1 ± 1.9	14.5 ± 3.2	0.001^a^, < 0.001^b, c^
CNR	4.5 ± 4.4	4.8 ± 2.4	6.1 ± 2.8	1.000^a^, 0.094^b^, < 0.001^c^
ADC_pons_ (×10^-6^ mm^2^/s)	679.5 ± 46.2	674.5 ± 47.8	679.4 ± 35.9	0.779^a^, 1.000^b^, 0.959^c^
ADC_PitNET/pituitary adenoma_ (×10^-6^ mm^2^/s)	695.7 ± 141.8	701.3 ± 138.4	711.9 ± 134.5	1.000^a^, 0.359^b^, 0.309^c^

Data are presented as the mean ± standard deviation

^a^AP-DWI *versus* B_0_-corrected-DWI

^b^AP-DWI *versus* RDC-DWI

^c^B_0_-corrected-DWI *versus* RDC-DWI

*ADC* Apparent diffusion coefficient, *ADC*_*PitNET/pituitary adenoma*_ ADC value for the solid portion of pituitary neuroendocrine tumor (PitNET)/pituitary adenoma, *ADC*_*pons*_ ADC value for the pons, *AP* Anterior-posterior, *CNR* Contrast-to-noise ratio, *DWI* Diffusion-weighted imaging, *SNR* Signal-to-noise ratio, *PitNET* Pituitary neuroendocrine tumor, *RDC-DWI* Reverse encoding distortion correction DWI

### Phantom study

The *b* = 1,000 s/mm^2^ images on RDC-DWI showed the fewest artifacts and least distortion among the three DWIs (AP-DWI, B_0_-corrected-DWI, and RDC-DWI) (Fig. 4b, c, and d). ADC values measured in each of the five PVP concentrations showed no significant difference among the three DWIs for concentrations of 0%, 30%, and 40% (Table 5). Statistically significant differences in ADC values were found at PVP concentrations of 10% and 20%; however, ADC values at these two concentrations on RDC-DWI were the closest to the theoretical ADC values provided by the manufacturer [25].

**Table 5:** ADC values (× 10^-6^ mm^2^/s) of PVP vials in the phantom study according to DWI type

%PVP	AP-DWI	B_0_-corrected-DWI	RDC-DWI	Ground truth	*p*-value
0%	1,128.4 ± 28.3	1,126.3 ± 30.4	1,123.4 ± 18.6	1,090 ± 9	0.763^a^, 0.196^b^, 1.000^c^
10%	848.3 ± 2.9	850.1 ± 8.1	832.0 ± 1.2	825 ± 8	1.000^a^, < 0.001^b, c^
20%	619.3 ± 9.1	624.7 ± 10.4	606.1 ± 10.6	598 ± 7	0.002^a^, < 0.001^b, c^
30%	427.8 ± 19.1	430.9 ± 23.4	428.4 ± 19.1	396 ± 5	0.299^a^, 1.000^b^, 0.674^c^
40%	256.0 ± 13.49	264.2 ± 23.4	259.3 ± 9.8	237 ± 6	0.165^a^, 0.057^b^, 0.896^c^

Data are presented as the mean ± standard deviation. The 50% PVP data were discarded because of air contamination

^a^AP-DWI *versus* B_0_-corrected-DWI

^b^AP-DWI *versus* RDC-DWI

^c^B_0_-corrected-DWI *versus* RDC-DWI

*ADC* Apparent diffusion coefficient, *AP* Anterior-posterior, *DWI* Diffusion-weighted imaging, *PVP* Polymerpolyvinylpyrrolidone, *RDC-DWI* Reverse encoding distortion correction DWI

## Discussion

The present study demonstrated that thin-slice EPI-based RDC-DWI with a novel on-console distortion correction technique enabled excellent visualization of the pituitary region and surrounding normal structures. Overall, RDC-DWI outperformed the other DWIs (B_0_-corrected-DWI and AP-DWI), exhibiting the best image quality in terms of susceptibility artifacts, geometric distortion, visualization of cranial nerves and cavernous sinus invasion, and overall tumor visualization. The excellent visualization on RDC-DWI results from its characteristic image acquisition and distortion correction. The on-console distortion correction technique of RDC-DWI is based on the *b* = 1,000 s/mm^2^ images with reverse phase-encoding directions after application of MPGs in three orthogonal directions, in addition to the *b* = 0 s/mm^2^ images with reverse phase-encoding directions. Therefore, RDC-DWI achieved combined correction of distortion due to *B*_0_ field inhomogeneity and of eddy current-induced distortion. Moreover, the on-console distortion correction of RDC-DWI is more feasible in clinical practice than the existing off-console distortion correction tools [17–20].

In addition to reduction in susceptibility artifacts and distortion, RDC-DWI produced high-contrast images and had the highest SNR among the three DWIs (AP-DWI, B_0_-corrected-DWI, and RDC-DWI) even with slice thickness of 1.2 mm, which was achieved by two-fold number of excitations, combining images with AP and PA phase-encoding directions. Measurement of precise SNR in DWI after application of distortion correction is complicated; in the present study, however, RDC-DWI provided images with high SNR, whereas in previous studies, no DWI technique has achieved SNR higher than that of conventional EPI-DWI [8, 10, 12, 26]. Furthermore, compared with previously reported DWI techniques with distortion reduction, slice thickness of 1.2 mm in RDC-DWI resulted in higher spatial resolution with distortion correction and enabled observation from multiple directions using multiplanar reconstruction of the pituitary lesion and surrounding normal structures [7, 8, 10–13].

We also assessed the accuracy of RDC-DWI for visualizing cavernous sinus invasion by PitNET/pituitary adenoma, which has not been evaluated previously on DWI. Scores for visualization of cavernous sinus invasion were significantly better for RDC-DWI and B_0_-corrected-DWI than for AP-DWI. Based on our results, RDC-DWI and B_0_-corrected-DWI might visualize cavernous sinus invasion well even without contrast medium [27–30]. With regard to postoperative cases in particular, RDC-DWI and B_0_-corrected-DWI can contribute to early detection and visualization of residual PitNET/pituitary adenoma in the cavernous sinus [5, 31]. Occasionally, residual PitNET/pituitary adenoma in the cavernous sinus can be difficult to assess on T2-weighted or contrast-enhanced T1-weighted images after surgery due to postoperative change [32]; however, RDC-DWI is better able to visualize residual PitNET/pituitary adenoma compared with other sequences because the venous pool of the cavernous sinus appears as a signal void on DWI.

Among the three DWIs, RDC-DWI showed the best visualization of both the trigeminal and right oculomotor nerves. This finding indicates that RDC-DWI had fewer susceptibility artifacts than B_0_-corrected-DWI, for the reason that susceptibility artifact is strongest where the cranial nerves pass near the paranasal sinuses. Anatomical visualization of the optic nerves was not superior on RDC-DWI, probably because the optic chiasma or optic nerves were compressed in more than half of the studied cases, and there might have been minimal air space between the optic nerves and the PitNET/pituitary adenomas.

The acquisition time for thin-slice RDC-DWI was 4:15 min:s. Scan times were also long in previously reported advanced DWI techniques for the pituitary region, such as line-scan DWI [8], periodically rotated overlapping parallel lines with enhanced reconstruction (PROPELLER)/BLADE DWI [9, 10], turbo-spin-echo (TSE) DWI [12, 13], and readout-segmented EPI-DWI [14, 15], although parameters such as slice thickness, number of slices, field of view, and number of excitations were different from RDC-DWI. Scan time is longer for RDC-DWI than conventional EPI-DWI but is clinically acceptable considering the clinical usefulness of RDC-DWI in the pituitary region, as shown in this study. Distortion correction techniques with deep learning reconstruction might have potential to reduce scan time and improve image quality, although its usefulness and advantages remain unclear [33].

Our study has several limitations. First, we included images in both pre- and post-operative states. RDC-DWI seemed to have the best visualization both preoperatively and postoperatively, although we did not conduct a comparison. Second, we used 3D-T2WI as the reference in evaluating visualization of cavernous sinus invasion. Cavernous sinus invasion is generally diagnosed with contrast-enhanced T1WI [27, 28, 30, 32]; however, many of the present patients did not undergo contrast-enhanced MRI. We referred to the clinical course and surgical records when it was difficult to determine the diagnosis using only 3D-T2WI. Finally, we included relatively considerable number of patients; however, more pre- and postoperative patients may be needed to generalize our results. Further evaluation in other pituitary lesions is also desirable. In addition, in our phantom study, the 50% PVP data were discarded due to air contamination. The other phantom study on RDC-DWI seems more reliable [22].

In conclusion, thin-slice RDC-DWI provided excellent image quality in terms of distortion, susceptibility artifact, and visualization of tumor and surrounding normal structures. RDC-DWI achieved accurate and high-contrast visualization with high spatial resolution, using an on-console combined correction for distortion due to *B*_0_ field inhomogeneity and eddy current-induced distortion. Thin-slice RDC-DWI facilitates visualization of the pituitary region and surrounding normal structures even without contrast medium.

### Supplementary Information

**Additional file 1: Supplementary Table S1.** Interrater reliability of qualitative evaluations. **Supplementary Figure S1.** Image assessment criteria for susceptibility artifacts. Arrows indicate susceptibility artifacts. The slice with the strongest artifacts is evaluated. Score 0 (very poor), artifacts at almost all edges of the frontal or temporal lobe. Score 1 (poor), many linear artifacts (a and d). Score 2 (fair), some linear artifacts (b and e). Score 3 (good), spotty artifacts or artifacts with lower signal intensity compared with typical signal pileup artifacts (c and f). Score 4 (excellent), no artifacts. **Supplementary Figure S2.** Criteria for image assessment of cranial nerves. Arrows indicate signal loss along the cranial nerves. Score 0 (very poor), no anatomical visualization of the nerve. Score 1 (poor), only a small portion of the nerve is seen (d, right arrowhead). Score 2 (fair), part of the course of the nerve is seen (d, left). Score 3 (good), most of the course of the nerve is seen (a, bilateral; b, bilateral; e, right). Score 4 (excellent), the whole course of the nerve is clearly delineated (c, bilateral; e, left; f, bilateral). **Supplementary Figure S3.** Image assessment criteria for visualization of cavernous sinus invasion. Coronal reconstructed *b* = 1,000 s/mm^2^ images of AP-DWI (b and f), B_0_-corrected-DWI (c and g), and RDC-DWI (d and h) are evaluated in comparison with the corresponding coronal reconstructed three-dimensional T2-weighted imaging (3D-T2WI) (a and e). Red lines indicate the medial and lateral walls of the internal carotid arteries. Score 0 (poor), PitNET/pituitary adenoma is not well visualized in the cavernous sinus (b and f). Score 1 (fair), PitNET/pituitary adenoma is seen in the cavernous sinus, but the diagnosis of cavernous sinus invasion differs between DWI and 3D-T2WI. Score 2 (good), PitNET/pituitary adenoma is seen clearly in the cavernous sinus on DWI and the diagnosis of cavernous sinus invasion is the same on DWI and 3D-T2WI (c, d, g, and h, arrows). **Supplementary Figure S4.** Image assessment criteria for overall tumor visualization. Axial *b* = 1,000 s/mm^2^ images of AP-DWI (b), B_0_-corrected-DWI (c), and RDC-DWI (d) are evaluated in comparison with the corresponding axial reconstructed 3D-T2WI (a). Score 0 (very poor), tumor is not seen. Score 1 (poor), only a portion of the PitNET/pituitary adenoma is seen. Score 2 (fair), the PitNET/pituitary adenoma is seen, but its shape is distorted (b, arrow). Score 3 (good), most of the PitNET/pituitary adenoma is clearly seen, but the image is degraded by distortion (arrow) and blurring around the sphenoid sinus (arrowheads) (c). Score 4 (excellent), the PitNET/pituitary adenoma is clearly seen (d). **Supplementary Figure S5.** Example showing regions of interest (ROIs) placement. A ROI (yellow oval) is placed on the pons on AP-DWI (a), B_0_-corrected-DWI (b), and RDC-DWI (c); and also on the solid portion of the PitNET/pituitary adenoma on AP-DWI (d), B_0_-corrected-DWI (e), and RDC-DWI (f). **Supplementary Figure S6.** Histograms of qualitative evaluations. Susceptibility artifacts in the frontal and temporal lobes, anatomical visualization of cranial nerves, and overall tumor visualization are assessed using a 5-point Likert scale. Visualization of cavernous sinus invasion is assessed using a 3-point Likert scale. Image quality scores are compared among the three DWIs (AP-DWI, B_0_-corrected-DWI, and RDC-DWI) using the Friedman test followed by pairwise comparisons with Bonferroni correction. **Supplementary Figure S7.** A 79-year-old woman with unoperated clinically non-functioning PitNET/pituitary adenoma. Sagittal three-dimensional T2-weighted imaging (3D-T2WI) (a) and the corresponding reconstructed sagittal *b* = 1,000 s/mm^2^ images of AP-DWI (b), B_0_-corrected-DWI (c), and RDC-DWI (d) are shown. The 3D-T2WI shows the typical appearance of PitNET/pituitary adenoma (a). Severe geometric distortion, susceptibility artifacts, and signal pileup near the sphenoid sinus are seen on AP-DWI (b, arrows). There is less distortion on the B_0_-corrected-DWI; however, abnormal signal remains (c, arrows), along with blurring near the sphenoid sinus (c, arrowheads). Among the three DWIs, image quality is the best for RDC-DWI, which has the least geometric distortion and fewest susceptibility artifacts (d).

## Data Availability

Data generated or analyzed during the study are available from the corresponding author by request.

## Abbreviations

3D-T2WI: Three-dimensional T2-weighted imaging
ADC: Apparent diffusion coefficient
AP: Anterior-posterior
CNR: Contrast-to-noise ratio
DWI: Diffusion-weighted imaging
EPI: Echo-planar imaging
MPG: Motion probing gradient
PA: Posterior-anterior
PitNET: Pituitary neuroendocrine tumor
PVP: Polymerpolyvinylpyrrolidone
RDC-DWI: Reverse encoding distortion correction DWI
ROI: Region of interest
SNR: Signal-to-noise ratio
