# Supplementary material for: Thin-slice reverse encoding distortion correction DWI facilitates visualization of non-functioning pituitary neuroendocrine tumor (PitNET)/pituitary adenoma and surrounding normal structures
Source: Eur Radiol Exp. 2024 Mar 7;8:28. doi: 10.1186/s41747-024-00430-8 (PMC10917724; doi:10.1186/s41747-024-00430-8)
Supplement: Supplementary file 1 — Additional file 1: Supplementary Table S1. Interrater reliability of qualitative evaluations. Supplementary Figure S1. Image assessment criteria for susceptibility artifacts. Arrows indicate susceptibility artifacts. The slice with the strongest artifacts is evaluated. Score 0 (very poor), artifacts at almost all edges of the frontal or temporal lobe. Score 1 (poor), many linear artifacts (a and d). Score 2 (fair), some linear artifacts (b and e). Score 3 (good), spotty artifacts or artifacts with lower signal intensity compared with typical signal pileup artifacts (c and f). Score 4 (excellent), no artifacts. Supplementary Figure S2. Criteria for image assessment of cranial nerves. Arrows indicate signal loss along the cranial nerves. Score 0 (very poor), no anatomical visualization of the nerve. Score 1 (poor), only a small portion of the nerve is seen (d, right arrowhead). Score 2 (fair), part of the course of the nerve is seen (d, left). Score 3 (good), most of the course of the nerve is seen (a, bilateral; b, bilateral; e, right). Score 4 (excellent), the whole course of the nerve is clearly delineated (c, bilateral; e, left; f, bilateral). Supplementary Figure S3. Image assessment criteria for visualization of cavernous sinus invasion. Coronal reconstructed b = 1,000 s/mm2 images of AP-DWI (b and f), B0-corrected-DWI (c and g), and RDC-DWI (d and h) are evaluated in comparison with the corresponding coronal reconstructed three-dimensional T2-weighted imaging (3D-T2WI) (a and e). Red lines indicate the medial and lateral walls of the internal carotid arteries. Score 0 (poor), PitNET/pituitary adenoma is not well visualized in the cavernous sinus (b and f). Score 1 (fair), PitNET/pituitary adenoma is seen in the cavernous sinus, but the diagnosis of cavernous sinus invasion differs between DWI and 3D-T2WI. Score 2 (good), PitNET/pituitary adenoma is seen clearly in the cavernous sinus on DWI and the diagnosis of cavernous sinus invasion is the same on DWI and 3D [file 41747_2024_430_MOESM1_ESM.docx]

**Thin-slice reverse encoding distortion correction DWI facilitates visualization of non-functioning pituitary neuroendocrine tumor (PitNET)/pituitary adenoma and surrounding normal structures**

**ELECTRONIC SUPPLEMENTARY MATERIAL**

**Supplementary Table S1**

Interrater reliability of qualitative evaluations

|  | κ |
| --- | --- |
| Frontal artifact | 0.59 |
| Temporal artifact | 0.66 |
| Right optic nerve | 0.61 |
| Left optic nerve | 0.64 |
| Right oculomotor nerve | 0.78 |
| Left oculomotor nerve | 0.71 |
| Right trigeminal nerve | 0.66 |
| Left trigeminal nerve | 0.74 |
| Cavernous sinus invasion | 0.69 |
| Overall tumor visualization | 0.76 |

The calculated κ statistic is interpreted as follows: ≤ 0.20, slight agreement; 0.21–0.40, fair agreement; 0.41–0.60, moderate agreement; 0.61–0.80, substantial agreement; and 0.81–1.00, almost perfect agreement.

**Supplementary Figure S1**

**
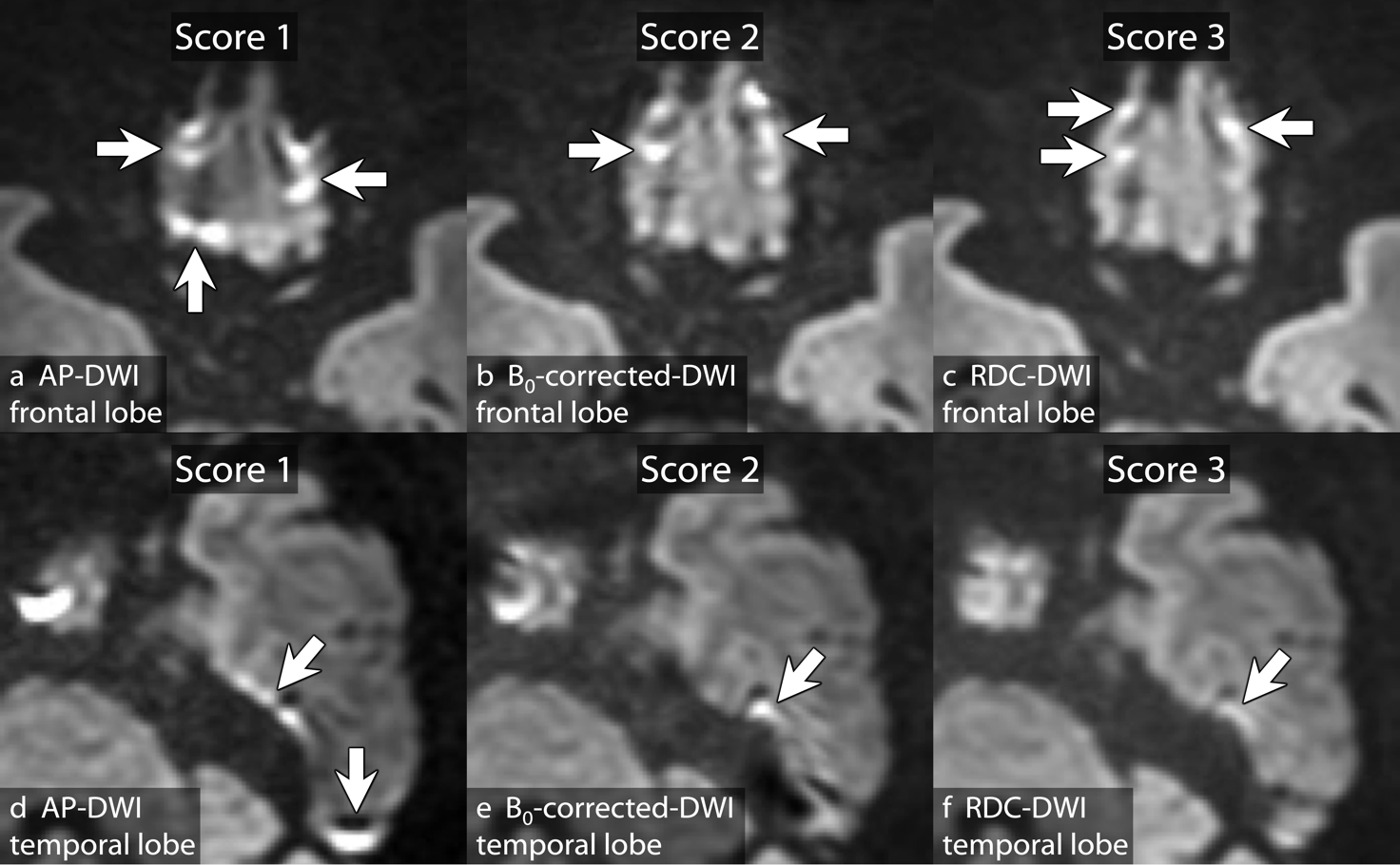
**

Image assessment criteria for susceptibility artifacts. Arrows indicate susceptibility artifacts. The slice with the strongest artifacts is evaluated. Score 0 (very poor), artifacts at almost all edges of the frontal or temporal lobe. Score 1 (poor), many linear artifacts (**a** and **d**). Score 2 (fair), some linear artifacts (**b** and **e**). Score 3 (good), spotty artifacts or artifacts with lower signal intensity compared with typical signal pileup artifacts (**c** and **f**). Score 4 (excellent), no artifacts.

**Supplementary Figure S2**

**
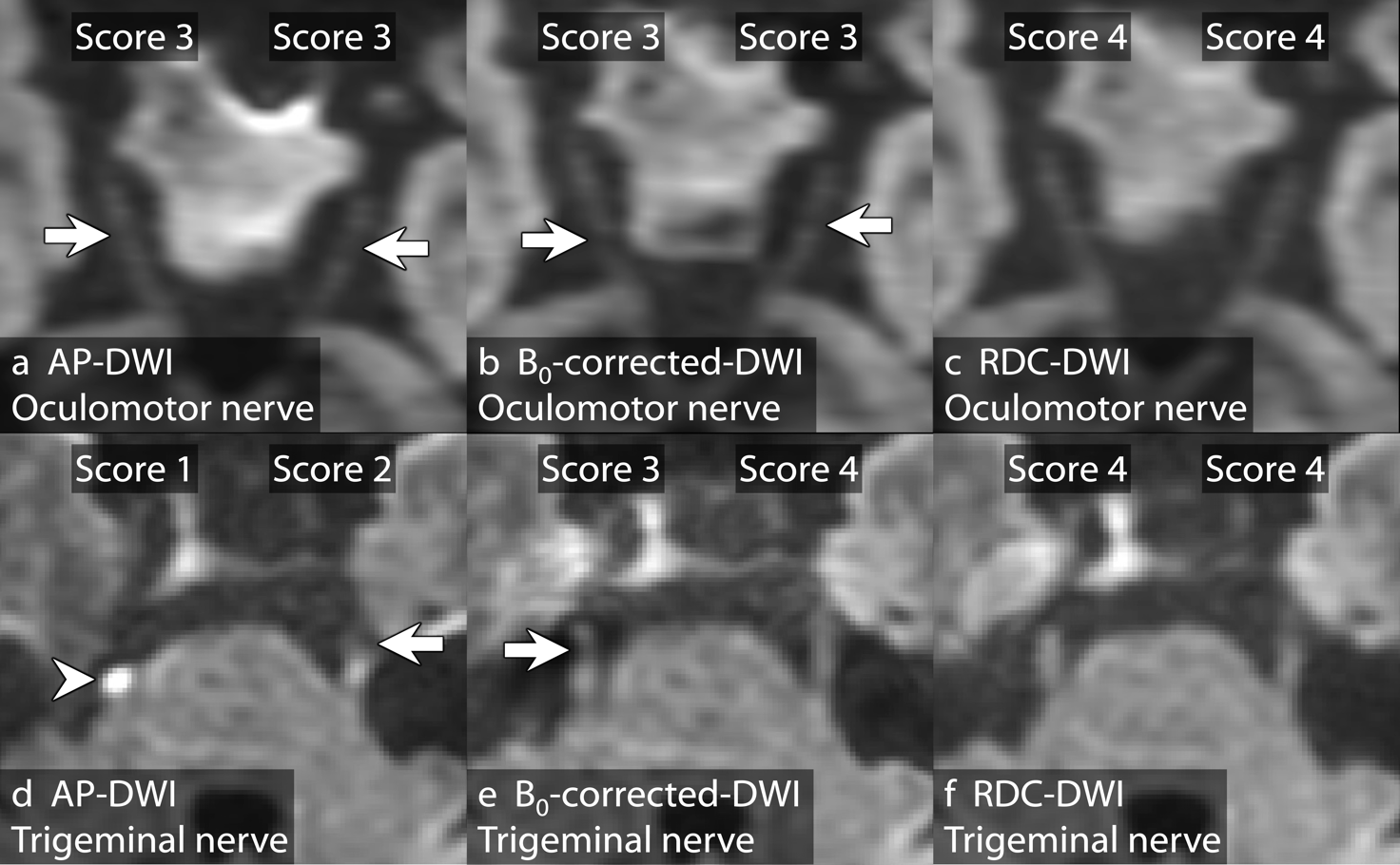
**

Criteria for image assessment of cranial nerves. Arrows indicate signal loss along the cranial nerves. Score 0 (very poor), no anatomical visualization of the nerve. Score 1 (poor), only a small portion of the nerve is seen (**d**, right arrowhead). Score 2 (fair), part of the course of the nerve is seen (**d**, left). Score 3 (good), most of the course of the nerve is seen (**a**, bilateral; **b**, bilateral; **e**, right). Score 4 (excellent), the whole course of the nerve is clearly delineated (**c**, bilateral; **e**, left; **f**, bilateral).

**Supplementary Figure S3**

**
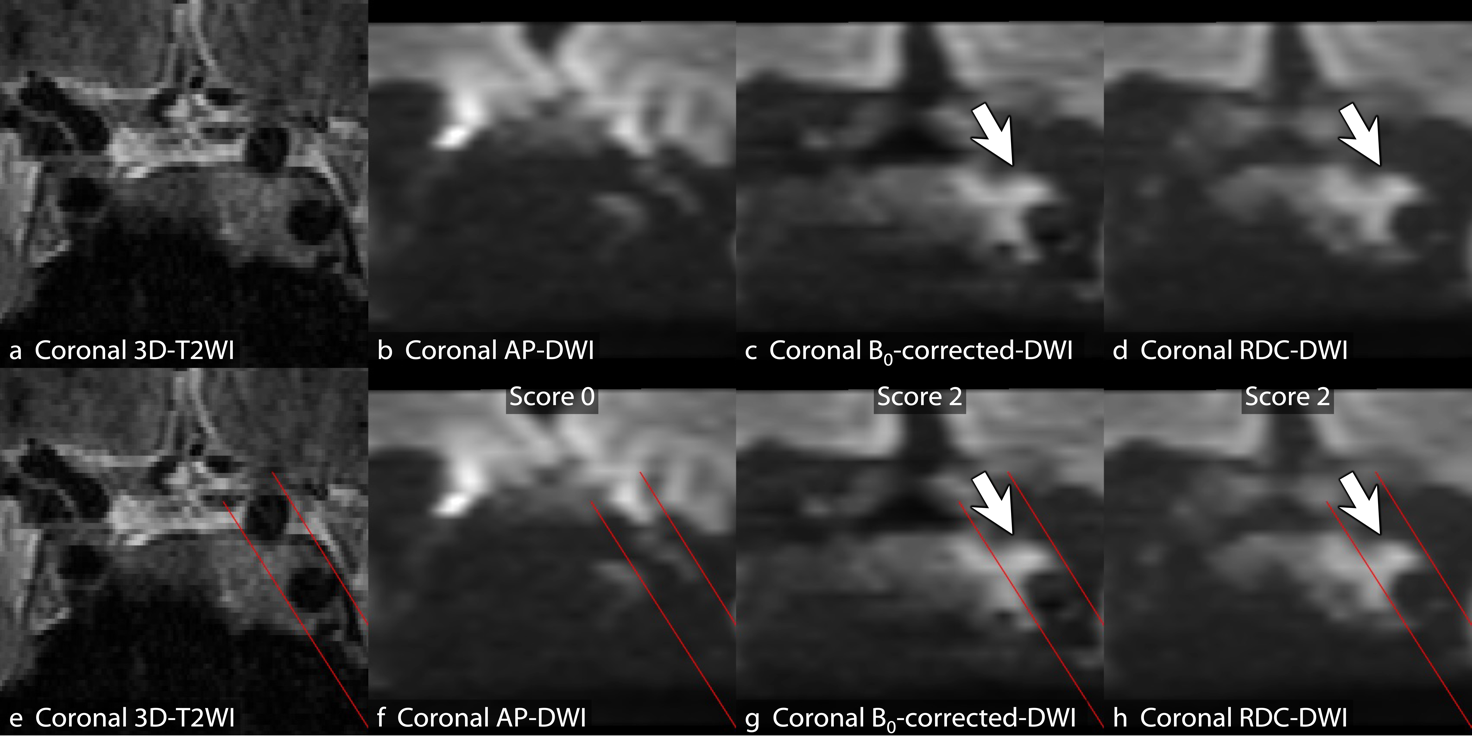
**

Image assessment criteria for visualization of cavernous sinus invasion. Coronal reconstructed b = 1,000 s/mm^2^ images of AP-DWI (**b** and **f**), B_0_-corrected-DWI (**c** and **g**), and RDC-DWI (**d** and **h**) are evaluated in comparison with the corresponding coronal reconstructed three-dimensional T2-weighted imaging (3D-T2WI) (**a** and **e**). Red lines indicate the medial and lateral walls of the internal carotid arteries. Score 0 (poor), PitNET/pituitary adenoma is not well visualized in the cavernous sinus (**b** and **f**). Score 1 (fair), PitNET/pituitary adenoma is seen in the cavernous sinus, but the diagnosis of cavernous sinus invasion differs between DWI and 3D-T2WI. Score 2 (good), PitNET/pituitary adenoma is seen clearly in the cavernous sinus on DWI and the diagnosis of cavernous sinus invasion is the same on DWI and 3D-T2WI (**c**, **d**, **g**, and **h**, arrows).

**Supplementary Figure S4**

**
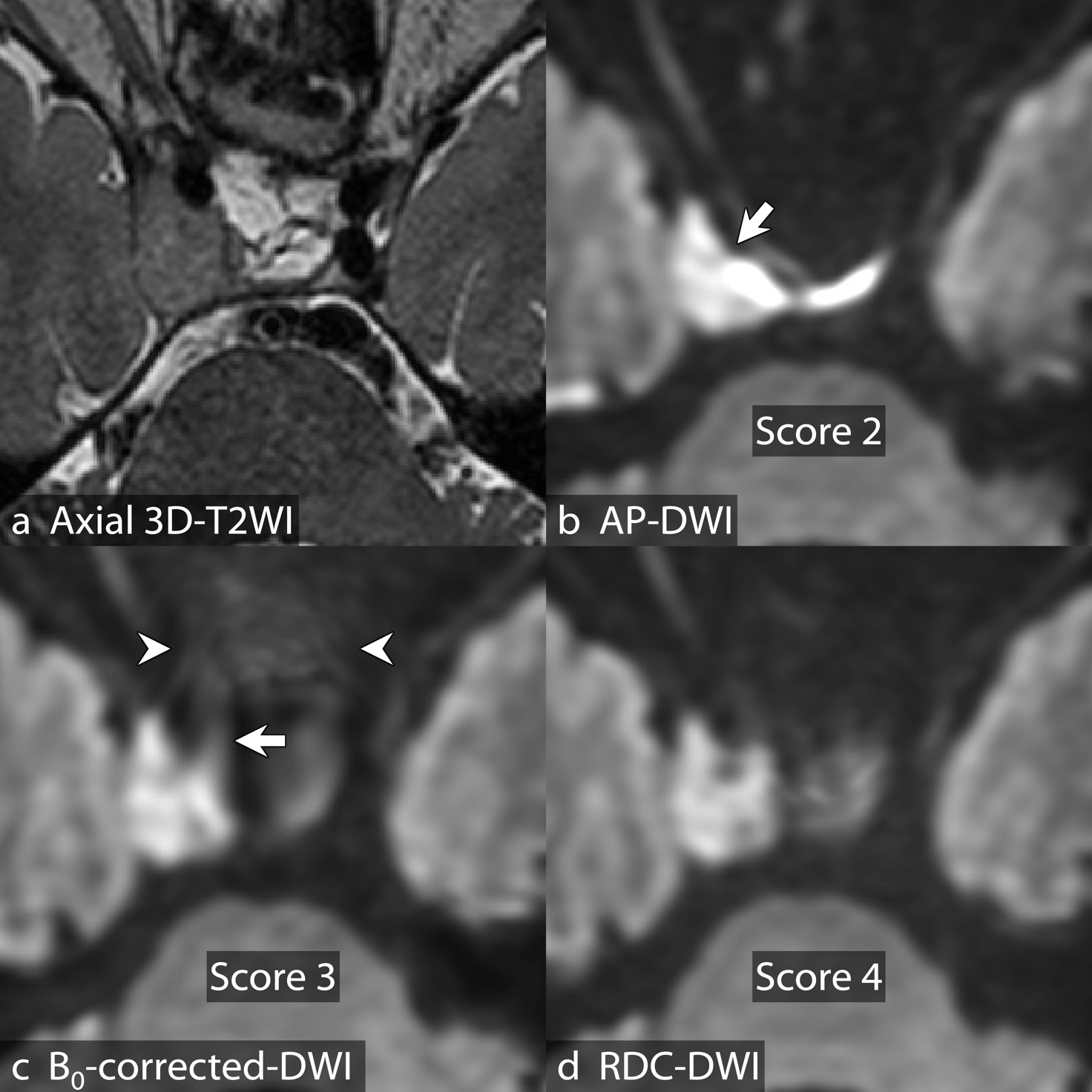
**

Image assessment criteria for overall tumor visualization. Axial b = 1,000 s/mm^2^ images of AP-DWI (**b**), B_0_-corrected-DWI (**c**), and RDC-DWI (**d**) are evaluated in comparison with the corresponding axial reconstructed 3D-T2WI (**a**). Score 0 (very poor), tumor is not seen. Score 1 (poor), only a portion of the PitNET/pituitary adenoma is seen. Score 2 (fair), the PitNET/pituitary adenoma is seen, but its shape is distorted (**b**, arrow). Score 3 (good), most of the PitNET/pituitary adenoma is clearly seen, but the image is degraded by distortion (arrow) and blurring around the sphenoid sinus (arrowheads) (**c**). Score 4 (excellent), the PitNET/pituitary adenoma is clearly seen (**d**).

**Supplementary Figure S5**

**
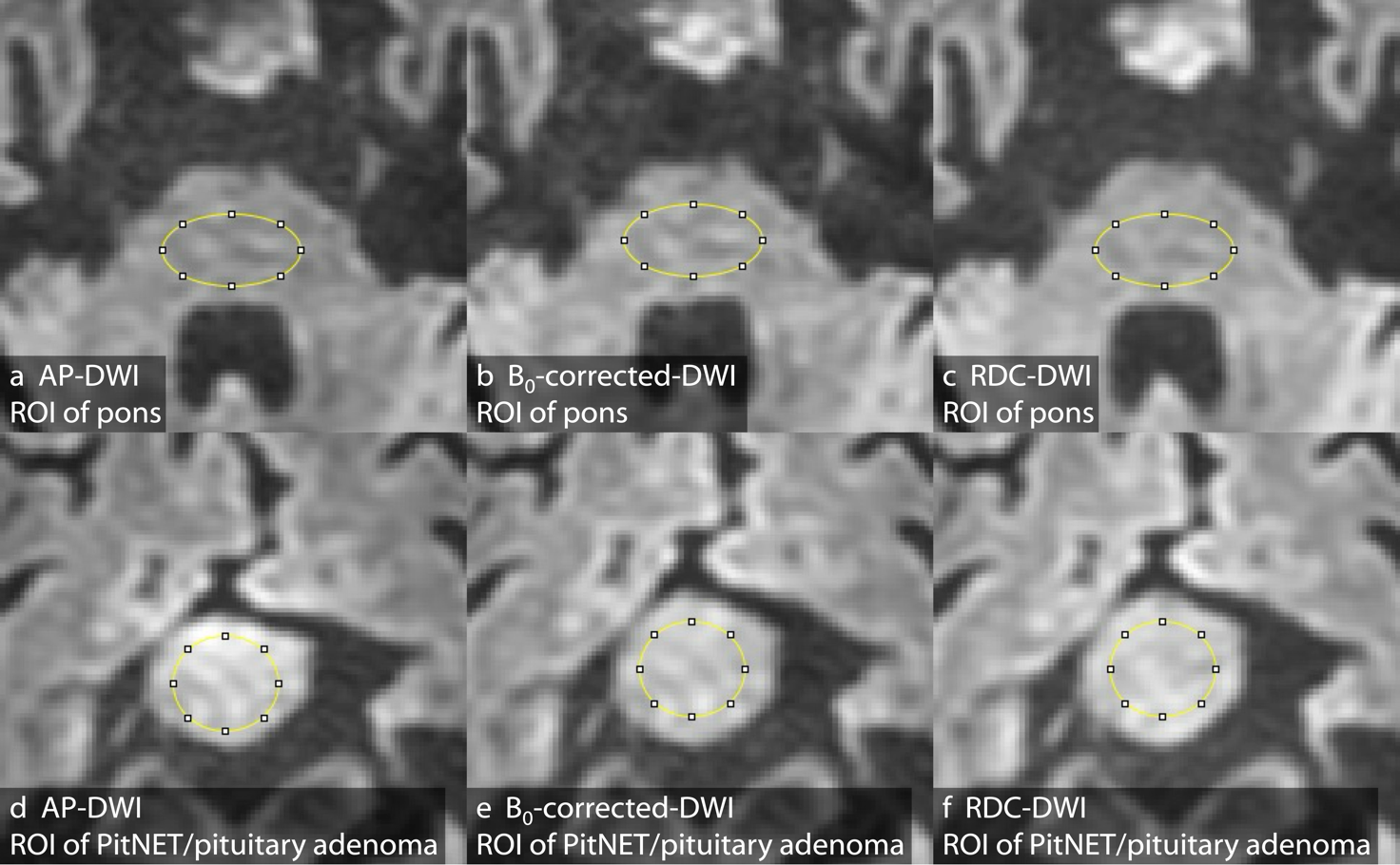
**

Example showing regions of interest (ROIs) placement. A ROI (yellow oval) is placed on the pons on AP-DWI (**a**), B_0_-corrected-DWI (**b**), and RDC-DWI (**c**); and also on the solid portion of the PitNET/pituitary adenoma on AP-DWI (**d**), B_0_-corrected-DWI (**e**), and RDC-DWI (**f**).

**Supplementary Figure S6**

**
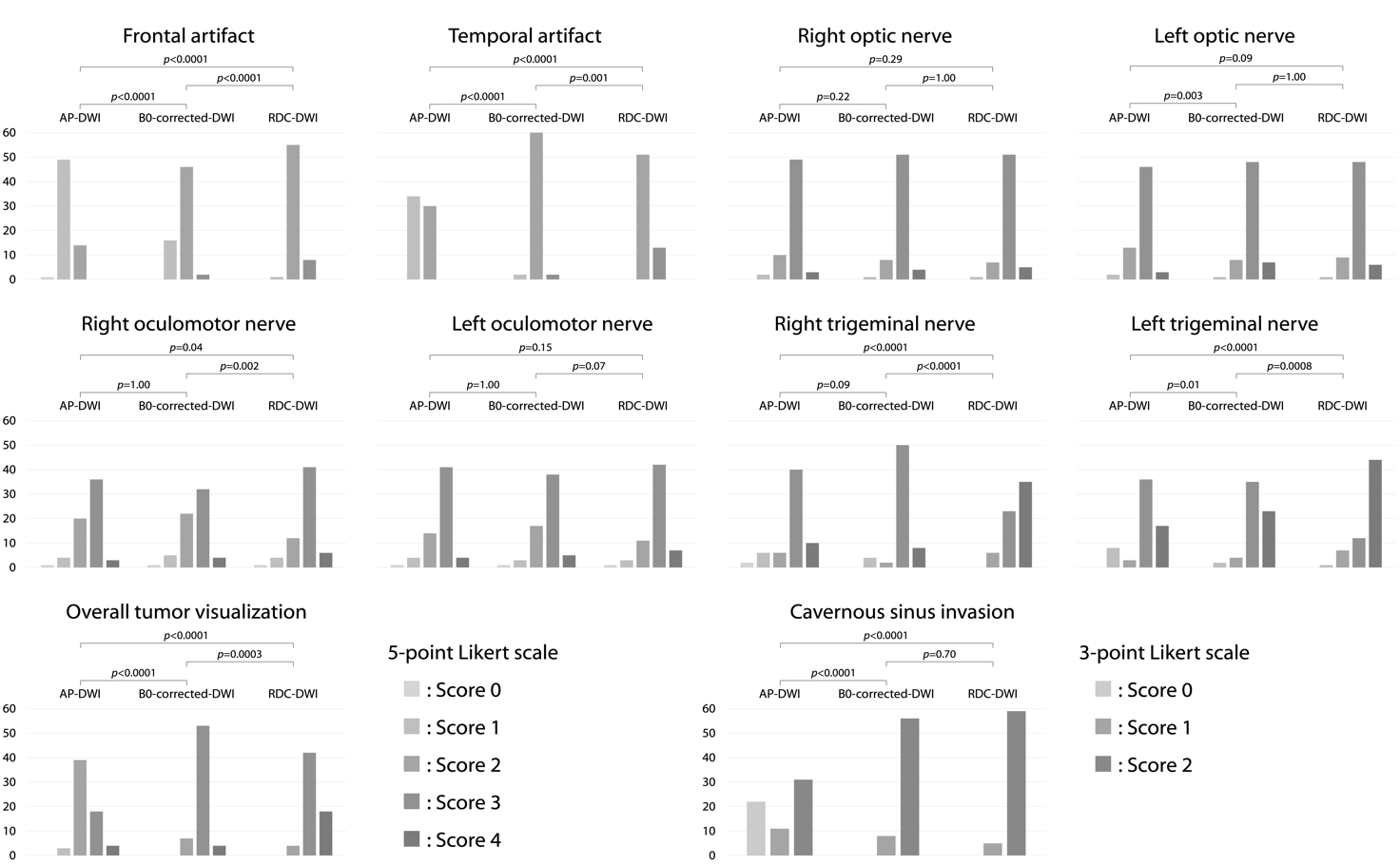
**

Histograms of qualitative evaluations. Susceptibility artifacts in the frontal and temporal lobes, anatomical visualization of cranial nerves, and overall tumor visualization are assessed using a 5-point Likert scale. Visualization of cavernous sinus invasion is assessed using a 3-point Likert scale. Image quality scores are compared among the three DWIs (AP-DWI, B_0_-corrected-DWI, and RDC-DWI) using the Friedman test followed by pairwise comparisons with Bonferroni correction.

**Supplementary Figure S7**


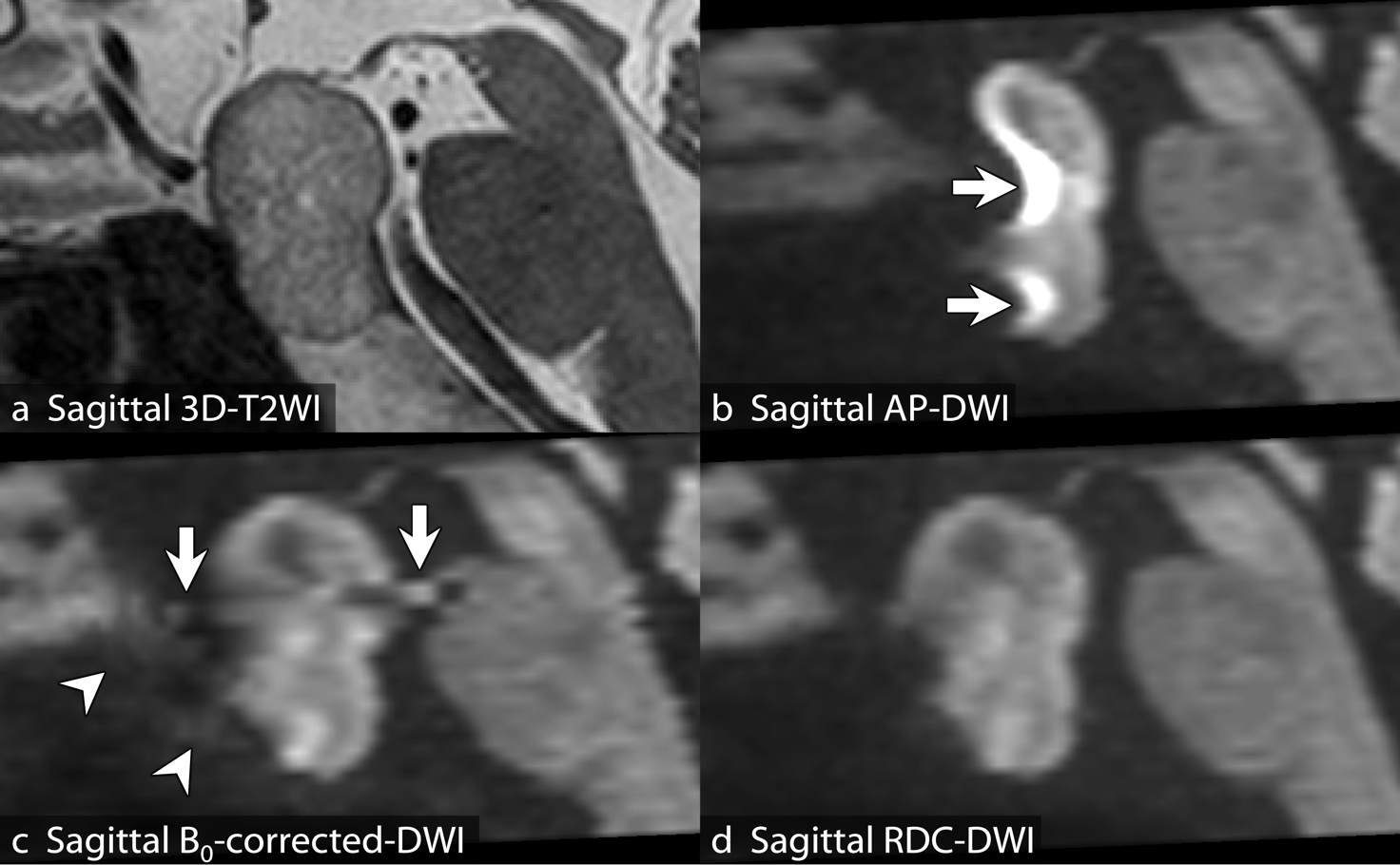


A 79-year-old woman with unoperated clinically non-functioning PitNET/pituitary adenoma. Sagittal three-dimensional T2-weighted imaging (3D-T2WI) (**a**) and the corresponding reconstructed sagittal b = 1,000 s/mm^2^ images of AP-DWI (**b**), B_0_-corrected-DWI (**c**), and RDC-DWI (**d**) are shown. The 3D-T2WI shows the typical appearance of PitNET/pituitary adenoma (**a**). Severe geometric distortion, susceptibility artifacts, and signal pileup near the sphenoid sinus are seen on AP-DWI (**b**, arrows). There is less distortion on the B_0_-corrected-DWI; however, abnormal signal remains (**c**, arrows), along with blurring near the sphenoid sinus (**c**, arrowheads). Among the three DWIs, image quality is the best for RDC-DWI, which has the least geometric distortion and fewest susceptibility artifacts (**d**).
